# Serum homocysteine, vitamin B 12 and folic acid levels in different types of glaucoma

**DOI:** 10.1186/1471-2415-6-6

**Published:** 2006-02-23

**Authors:** Tongabay Cumurcu, Semsettin Sahin, Erdinc Aydin

**Affiliations:** 1 Gaziosmanpasa University, School of Medicine, Department of Ophthalmology, Tokat, Turkey; 2 Gaziosmanpasa University, School of Medicine, Department of Biochemistry, Tokat, Turkey

## Abstract

**Background:**

This study was performed to compare levels of serum homocysteine (Hcy), vitamin B12 and folic acid in patients with primary open-angle glaucoma (POAG), pseudoexfoliative glaucoma (PEXG), normotensive glaucoma (NTG) and healthy controls.

**Methods:**

Twentyfive patients with POAG, 24 with PEXG, and 18 with NTG, along with 19 control healthy subjects were included this prospective study. Levels of serum Hcy were measured using immunoassay, and those of serum vitamin B12 and folic acid were measured using competitive chemiluminescent enzyme immunoassay.

**Results:**

The mean Hcy concentration in the PEXG group was significantly higher (P < 0.001) as compared to the other groups. There were no significant differences with respect to the mean Hcy concentrations among other groups (P > 0.05). There were no statistical differences in serum vitamin B12 levels among POAG, PEXG, NTG and control subjects (P > 0.05).

The mean serum folic acid level was significantly lower in the subjects with PEXG (P < 0.009). However, the mean folic acid concentrations among the other groups did not differ significantly (P > 0.05).

**Conclusion:**

Elevated levels of Hcy in PEXG may explain the role of endothelial dysfunction among patients with PEXG.

## Background

Intraocular pressure (IOP) is assumed to be the most significant risk factor in glaucoma, but recent evidences indicate that vascular risk factors may also play a role. Impaired microcirculation and abnormal perfusion may cause glaucomatous damage in the optic nerve head. Anatomical or functional abnormalities of the vessels of the optic nerve head such as arteriosclerosis or vascular dysregulation might be the causative factor [1,2].

The metabolism of methionine via homocysteine (Hcy) to cysteine is a complex pathway involving an enzyme depending on vitamin B12, B6, and folic acid. Genetically inherited defects are the most important determinants of elevated levels of Hcy. Some studies showed that elevated Hcy may increase the risk of retinal vascular diseases, such as retinal artery and vein occlusion and non-artheritic ischemic optic neuropathy [3-7]. Hcy-induced vascular problem may be a multifactorial case, including direct toxic damage to the endothelium, stimulation of proliferation of smooth muscle cells, enhanced low density lipoprotein peroxidation, increased platelet aggregation, and effects upon the coagulation system [8].

According to one study, serum Hcy concentrations in patients with vascular disease are on average 30% higher than in normal subjects [9]. In addition, mild hyperhomocysteinemia might be a risk factor for myocardial infarction, stroke, abdominal aorta aneurysms and vascular dementia [10-13].

Recently, higher levels of plasma Hcy in primary open-angel glaucoma (POAG) and pseudoexfoliative glaucoma (PEXG) patients were reported [14-18]. On the other hand, some studies reported no significant differences in levels of plasma Hcy in POAG and normotensive glaucoma (NTG) [16,18,19].

The aim of this study is to evaluate the role of serum Hcy, vitamin B12 and folic acid levels in patients with POAG, PEXG, and NTG, and compare them to sex and age-matched group of control healthy subjects.

## Methods

Total of 25 patients with POAG, 24 with PEXG, and 18 with NTG, along with 19 control healthy subjects undergoing ocular surgery at the Gaziosmanpaşa University, Department of Ophthalmology between October 2004 and May 2005 were enrolled in this case control study. The study was conducted according to the tenets of the Declaration of Helsinki, and patients gave informed consent after the nature and intent of the study had been fully explained to them.

All patients underwent a complete ophthalmic examination, including visual acuity, slit-lamp examination, gonioscopy, tonometry, fundoscopy, visual field examination, and systemic examination.

Inclusion criteria were as follows:

1-POAG was defined by the presence of an open angle on gonioscopy, IOP ≥ 22 mmHg measured with a Goldman Applanation Tonometer, typical glaucomatous cupping and visual field defect in at least one eye on standard automated perimetry (full-threshold or Swedish Interactive Threshold Algorithm (SITA) strategy, program 24-2, Humphrey Field Analyzer).

2-Diagnosis of PEXG was based on the presence of typical exfoliation material on the anterior lens capsule in one or both eyes with typical glaucomatous cupping and visual field defect in at least one eye. IOP was ≥22 mmHg with an open angle in gonioscopic examination.

3-NTG patients were characterized by the presence of glaucomatous optic neuropathy and visual field defect in at least one eye and IOP was <22 mmHg.

4-Control subjects had no history of elevated IOP higher than 22 mmHg, no exfoliative material on the anterior lens capsule, normal visual fields and optic discs.

Exclusion criteria included non-genetic factors associated with hyperhomocysteinemia, a history of diabetes mellitus, systemic hypertension, peripheral or coronary artery disease, cerebrovascular disease, renal dysfunction, ocular inflammation, retinal occlusive disease. In addition, we excluded those taking vitamin and other medications known to affect Hcy measurements, such as phenytoin, methotrexate, vitamins B6, B12 and folic acid.

### Serum total hcy, vitamin B12 and folic acid analysis

A single 5-ml venous blood sample for serum Hcy, vitamin B12 and folic acid detection was collected in the fasting state. The samples were centrifuged within 1 h after collection and stored at -40°C. The levels of serum Hcy were measured using an immunoassay procedure (Immulite 2000-BIODPC, USA). This assay gives normal values of the serum Hcy as 12–15 μmol/l according to the manufacturer. The levels of serum vitamin B12 and folic acid were measured using competitive chemiluminescent enzyme immunoassay (Immulite 2000-BIODPC, USA). For these assays, the normal serum values of vitamin B12 and folic acid are, respectively, 193–982 pg/ml and 3–7 ng/ml, also according to manufacturer's instruction.

### Statistical analysis

Analysis of variance (one-way ANOVA) was used to detect differences in continuous variables (Hcy, vitamin B12 and folic acid, age) among the 4 study groups. For categorical variables (gender), chi-square tests was used to assess differences between groups. P value <0.05 was considered statistically significant. Statistical analyses were performed using software SPSS version 10.00.

## Results

There were no significant differences in gender and age between control group and the other groups (Table 1).

**Table 1:** Demographic data characteristics of the study groups. POAG: primary open-angel glaucoma PEXG: pseudoexfoliative glaucoma, NTG: normotensive glaucoma, SD: standard deviation.

	**POAG (n = 25)**	**PEXG (n = 24)**	**NTG (n = 18)**	**Control (n = 20)**	**P value**
Age range	44–78	48–79	50–71	50–67	0.71
Mean ± SD	56.76 ± 12.58	61.66 ± 10.05	57.77 ± 7.27	55.63 ± 4.04	
Male	7 (28%)	10 (41.7%)	6 (33.3%)	5 (26.3%)	0.686
Female	18 (72%)	14 (58.3%)	12 (66.7%)	14 (73.7%)	

Levene's variance homogeneity analysis showed that the variances of groups were homogenous in serum Hcy, vitamin B12, folic acid, age and gender (P > 0.05).

**Figure 1:**
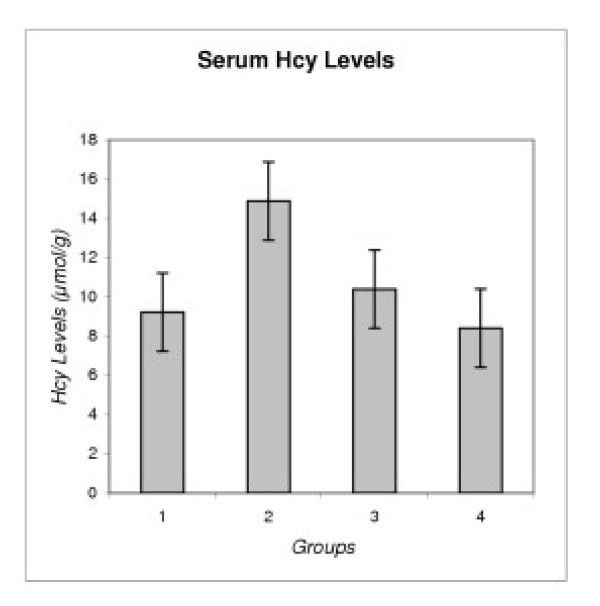
Serum Homocysteine Levels in Different Types of Glaucoma. Group 1: Primary Open-angel Glaucoma (POAG), Group 2: Pseudoexfoliative Glaucoma (PEXG), Group 3: Normotensive Glaucoma (NTG), Group 4: Control Group.

The parametric one-way ANOVA test indicated that the mean Hcy concentration in the PEXG group was significantly higher (P < 0.001) than in the other groups. Mean ± standard deviation (SD) of Hcy levels in PEXG was 14.88 ± 3.26 μmol/l. The mean Hcy concentrations among the other groups were not significantly different (P > 0.05). Mean ± SD of Hcy levels in POAG, NTG and control subjects were 9.22 ± 3.70, 10.39 ± 2.89, 8.40 ± 2.77 μmol/l, respectively (Table 2, and Figure 1).

**Table 2:** Serum homocysteine, vitamin B 12 and folic acid levels in different types of glaucoma.

Patient	Number	Hcy(μmol/g)	Vit.B12(pg/ml)	Folate(ng/ml)
PEXG	24	14.88 ± 3.26*	209.37 ± 104.44	4.26 ± 1.69*
POAG	25	9.22 ± 3.70	232.84 ± 67.55	6.24 ± 2.88
NTG	18	10.39 ± 2.89	262.33 ± 85.94	5.97 ± 1.85
Control	19	8.40 ± 2.77	261.84 ± 126.22	5.93 ± 1.70

*Statistically significant

**Figure 2:**
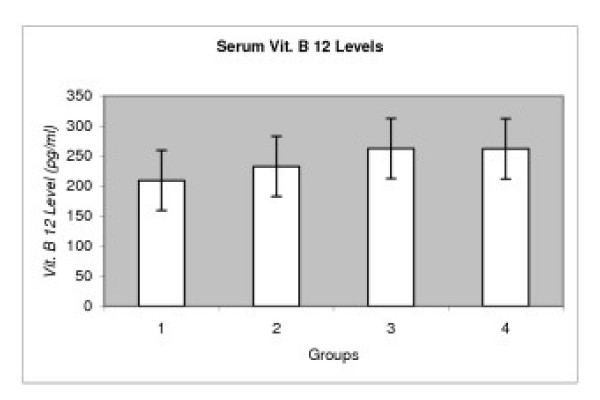
Serum Vit. B 12 Levels in Different Types of Glaucoma. Group 1:PEXG, Group 2: POAG, Group 3: NTG, Group 4: Control.

There were no statistical differences in serum vitamin B12 levels among POAG, PEXG, NTG and control subjects (P > 0.05). Mean ± SD of vitamin B12 levels in POAG, PEXG, NTG and control subjects were 209.37 ± 104.44, 232.84 ± 67.55, 262.33 ± 85.94, 261.84 ± 126.22 pg/ml, respectively (Table 2, and Figure 2).

**Figure 3:**
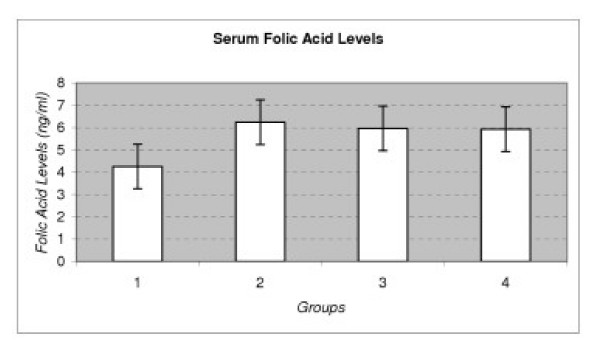
Serum Folic Acid Levels in Different Types of Glaucoma. Group 1:PEXG, Group 2: POAG, Group 3: NTG, Group 4: Control.

When all groups were compared, it has been found that the mean serum folic acid level was significantly lower in the subjects with PEXG (P < 0.009). Mean ± SD of folic acid levels in PEXG was 4.26 ± 1.69 ng/ml. The mean folic acid concentrations among the other groups were not significantly different (P > 0.05). Mean ± SD of folic acid levels in POAG, NTG and control subjects were 6.24 ± 2.88, 5.97 ± 1.85, 5.93 ± 1.70 ng/ml, respectively (Table 2, and Figure 3).

## Discussion

Some studies have demonstrated that Hcy induces overproduction of oxidative radicals which, in turn, causes intimal damage and activate serine elastase in arterial smooth muscle cells [8,20]. Activation of matrix metalloproteinase 2 causes elastolysis of elastin and fibrillar collagen in arterial media [20]. Oxidative stress may explain the effect of homocysteine in systemic vascular diseases. Folic acid is able to scavenge oxygen radicals and improves endothelial function [21]. Dietary folic acid (0.5–5 mg/day) reduces basal Hcy levels by 25% [22].

In the current study, we found elevated serum Hcy levels and reduced folic acid levels to be associated with PEXG when compared with the other groups. No statistically significant difference was found between plasma Hcy, folic acid and vitamin B12 levels in POAG, NTG and control patients.

Distribution circulation is involved in the pathogenesis of glaucomatous damage [1]. The endothelium plays an important role in the regulation of vascular tone. Hcy, being one of the important vasoconstructive factors, induces vascular oxidative stres [23]. PEX is also related with oxidative stres [24]. It is also known that homocysteine is a risc factor for atherosclerosis and chronic elevation of plasma homocysteine impairs endothelium-dependent vasodilatation [22]. The cause for this is an inhibition of the NO-synthesis by asymmetric dimethylarginine (ADMA). ADMA is increased by oxidative stress and also by homocysteine [25]. This events may explain the increased risk of vascular disease among patients with PEXG.

We observed significantly elevated Hcy levels in plasma of PEXG patients, which is in line with previous studies [15-18]. PEX materials are deposited in the adventia of the iris vessels associated with degeneration of endothelial cells. The iris vasculopathy causes blood-aqueous barrier dysfunction and anterior segment hypopxia [26]. Since PEX is a systemic disorder, this vasculopathy may also affect other tissues of the body.

An association of exfoliative syndrome with branch and central retinal vein occlusion has been suggested [27,28]. In the Blue Mountain Eye Study, exfoliation syndrome correlated positively with a history of hypertension, angina, myocardial infarction, or stroke [29]. In addition, some studies have reported a higher frequency of exfoliation syndrome in patients with abdominal aortic aneurysm and transient ischemic attacks [30,31].

Although previous studies have reported elevated plasma Hcy levels in PEXG, only Puustjarvi et al. measured erythrocyte folic acid, serum vitamin B 12 and B6 levels, and reported that the erythrocyte folic acid, serum vitamin B12 and B6 levels did not differ statistically between PEXG group and controls [15-18]. Our study demonstrates that decreased serum folic acid levels occur only in PEXG group, and that there was no significant difference in vitamin B12 level among PEXG, POAG, NTG and control groups. Further studies should be performed to demonstrate whether the ocular and systemic ischemic changes and neurodegeneration may be prevented or slowed down in PEXG with folic acid supplementation.

Only one study reported elevated serum Hcy level in POAG patients and suggested that thermolabile methylenetetrahydrofolate reductase deficiency may be in part the cause of the increased serum Hcy level in patient with POAG [14]. We found that serum Hcy level in patients with POAG did not statistically differ from the other groups. This finding is supported with the study of Altintas et al. [18] and Wang et al. [19].

Vessani et al. [16] suggested an elevated plasma Hcy level is more common in PEXG patients than in control subjects, but not in patients with NTG. We also find that serum Hcy levels were not significantly different from the other groups. In addition, there was no difference serum vitamin B 12 and folic acid levels in this group.

To the best of our knowledge, this is the first study that has compared Hcy, folic acid and vitamin B12 levels measured by the immunoassay method among patients with PEXG, POAG, NTG and control groups.

Hyperhomocysteinemia is related with vascular dysfunction, glaucoma and pseudoexfoliative syndrome, but whether it is as a cause or a consequence remains to be clarified. These results suggested that a relationship only with PEXG-associated systemic and ocular vasculopathy.

Further larger scale studies are needed to elucidate the role of hyperhomocysteinemia in different types of glaucoma due to evaluated levels of Hcy in plasma, ocular (aqueous humour or vitreous), and the other tissues.

## Conclusion

In our study, among various glaucoma types, only in PEXG type serum levels of Hcy was found to be increased and serum levels of folic acid was found to be decreased.

## List of abbreviations used

**Hcy: **homocysteine

**POAG: **primary open-angle glaucoma

**PEXG: **pseudoexfoliative glaucoma

**IOP: **intraocular pressure

**SITA: **Swedish Interactive Treshold Algorithm

**ADMA: **asimmetric dimethylarginine

## Competing interests

The author(s) declare that they have no competing interests.

## Pre-publication history

The pre-publication history for this paper can be accessed here:


